# Predation on *Drosophila suzukii* within Hedges in the Agricultural Landscape

**DOI:** 10.3390/insects12040305

**Published:** 2021-03-30

**Authors:** Alexandra Siffert, Fabian Cahenzli, Patrik Kehrli, Claudia Daniel, Virginie Dekumbis, Barbara Egger, Jana Furtwengler, Camille Minguely, Nicola Stäheli, Franco Widmer, Dominique Mazzi, Jana Collatz

**Affiliations:** 1 Research Division Agroecology and Environment, Agroscope, Reckenholzstrasse 191, 8046 Zürich, Switzerland; alexandra.siffert@uzh.ch; 2 Institute of Plant and Microbial Biology, University of Zurich, Zollikerstrasse 107, 8008 Zürich, Switzerland; 3 Department of Crop Sciences, FiBL, Research Institute of Organic Agriculture, Ackerstrasse 113, 5070 Frick, Switzerland; fabian.cahenzli@fibl.org (F.C.); claudia.daniel@fibl.org (C.D.); jana.furtwengler@fibl.org (J.F.); 4 Research Division Plant Protection, Agroscope, Route de Duillier 50, 1260 Nyon, Switzerland; patrik.kehrli@agroscope.admin.ch; 5 Research Division Plant Protection, Agroscope, Route des Eterpys 18, 1964 Conthey, Switzerland; virginie.dekumbis@agroscope.admin.ch (V.D.); camilleolivia.minguely@bfh.ch (C.M.); 6 Competence Division Plants and Plant Products, Agroscope, Müller-Thurgau-Strasse 29, 8820 Wädenswil, Switzerland; barbara.egger@agroscope.admin.ch (B.E.); nicola.staeheli@agroscope.admin.ch (N.S.); 7 School of Agricultural, Bern University of Applied Sciences, Forest and Food Sciences HAFL, Länggasse 85, 3052 Zollikofen, Switzerland; 8 Molecular Ecology, Agroscope, Reckenholzstrasse 191, 8046 Zürich, Switzerland; franco.widmer@agroscope.admin.ch; 9 Research Division Plant Protection, Agroscope, Müller-Thurgau-Strasse 29, 8820 Wädenswil, Switzerland; dominique.mazzi@agroscope.admin.ch

**Keywords:** biological control, predators, molecular gut content analysis, earwigs, spiders, predatory bugs, semi-natural habitat

## Abstract

**Simple Summary:**

*Drosophila suzukii* is an invasive species that feeds and reproduces on various cultivated and wild fruits and moves between agricultural and semi-natural habitats, such as hedges and forest patches. These semi-natural habitats are known to harbor a diverse community of natural enemies of pests. When we exposed *D. suzukii* pupae in dry and humid hedges, we found that on average 44% of them were predated within four days. The most common predators in the hedges were earwigs, spiders, and ants. Using a molecular assay that detects the DNA of *D. suzukii* in the gut of predators, we could show that 3.4% of the sampled earwigs, 1.8% of the spiders, and one predatory bug had fed on *D. suzukii*. This small proportion may be due to methodological constraints. However, the overall predation rate helps to reduce *D. suzukii* populations, in particular in hedges that are scarce of host fruits.

**Abstract:**

The invasive *Drosophila suzukii* feeds and reproduces on various cultivated and wild fruits and moves between agricultural and semi-natural habitats. Hedges in agricultural landscapes play a vital role in the population development of *D. suzukii*, but also harbor a diverse community of natural enemies. We investigated predation by repeatedly exposing cohorts of *D. suzukii* pupae between June and October in dry and humid hedges at five different locations in Switzerland. We sampled predator communities and analyzed their gut content for the presence of *D. suzukii* DNA based on the COI marker. On average, 44% of the exposed pupae were predated. Predation was higher in dry than humid hedges, but did not differ significantly between pupae exposed on the ground or on branches and among sampling periods. Earwigs, spiders, and ants were the dominant predators. Predator communities did not vary significantly between hedge types or sampling periods. DNA of *D. suzukii* was detected in 3.4% of the earwigs, 1.8% of the spiders, and in one predatory bug (1.6%). While the molecular gut content analysis detected only a small proportion of predators that had fed on *D. suzukii*, overall predation seemed sufficient to reduce *D. suzukii* populations, in particular in hedges that provide few host fruit resources.

## 1. Introduction

The invasive, frugivorous *Drosophila suzukii* Matsumura (Diptera: Drosophilidae) oviposits into ripening cherries, berries, and grapes, causing large economic damage [1,2]. Besides these agricultural crops, *D. suzukii* infests more than 100 species of wild fruits, many of them commonly found in agricultural hedges [3,4,5]. Therefore, this highly mobile species may use fruits in hedges for reproduction when host crops are not available. Hedges in the agricultural landscape may consequently provide a source for the re-infestation of crops after pest management interventions. *Drosophila suzukii* further uses hedges and woodland as a refuge during unfavorably hot and dry weather in summer [6] and as an overwintering habitat [7]. In addition, other polyphagous pests, for example aphids, are known to utilize the diverse resources and shelter afforded by semi-natural habitats [8,9]. Several studies have shown that nearby hedges and forest promote *D. suzukii* in crops [10,11,12]. In a recent study, it was observed that a greater proportion of non-cropping habitat promoted larger populations of *D. suzukii*, whereas fewer generalist predators were captured in those landscapes [13]. However, many natural enemies that provide biological control of agricultural pests also respond positively to landscape complexity, such as semi-natural habitats between agricultural plots [14,15]. Hedges, for example, not only connect natural and semi-natural habitats with crops [16], but they can also be the source of biocontrol functions in agricultural landscapes [17] by satisfying natural enemy needs not covered by crops [18]. For instance, the microclimate within hedges is buffered compared to open fields and therefore offers shelter during adverse climatic conditions [17,19]. Hedges may also form refuges for natural enemies, as they are usually not sprayed with insecticides [20]. Furthermore, diverse hedges can provide food and alternative hosts when target pests are scarce in the crop [21].

Thus, hedges support a diverse fauna of natural enemies, among them generalist predators such as carabids, staphylinids, earwigs, spiders, and predatory bugs [17,22]. It has been shown that these generalist predators can provide baseline control of agricultural pests [23]. Some of them are known to prey on *D. suzukii* and may thus reduce its abundance [22,24,25]. We aimed to gain further insight into the role of generalist predators in hedges in agricultural landscapes for the control of *D. suzukii*. We exposed pupae of *D. suzukii* in dry and humid hedges at different locations in Switzerland during the growing season (June–October) and determined predation rates, assessed the community of arthropod predators within these hedges, and analyzed the gut content of captured predators with *D. suzukii*-specific primers in order to confirm their feeding on this prey.

## 2. Materials and Methods

### 2.1. Field Sites

Ten hedges embedded in the agricultural landscape were chosen at five different locations in Switzerland. Hedges were selected according to the known presence of *D. suzukii* from surveys conducted in previous years and all hedges contained a variety of potential host plants of this vinegar fly. One humid and one dry hedge were assessed at each location to cover a wide diversity of predator species. Dry hedges were sun exposed, whereas humid hedges were at shady locations, often close to a water course. Locations and plant species of each hedge are shown in Table 1.

### 2.2. Exposure of D. suzukii Pupae in the Field

Laboratory rearing of *D. suzukii* were established in the infrastructures of the authors’ affiliations, at Agroscope and FiBL. Adult flies were kept on an artificial banana-based diet (for a detailed recipe, see Boycheva et al. [26]) for oviposition. The diet containing eggs was replaced every 2–3 days, stored in ventilated jars, and kept for preparation of sample pupae or emergence of adult flies. For sample preparation, tissue paper was placed into the jars with last instar larvae for 24 h. During this time, larvae crawled onto the paper and pupated. The paper was then cut to contain the desired number of pupae for experiments. Rearing was conducted in climate chambers at 22 ± 1 °C, 70–75% RH and 16:8 L:D.

Exposure of pupae in the hedges took place at each location simultaneously in both hedge types at intervals of about one month between June and October 2019, accounting for four sampling periods: First: 17 June–4 July; Second: 5 July–26 July; Third: 12 August–2 September; Fourth: 17 September–18 October (Table 1). Three sets of thirty pupae per exposure period and per hedge were prepared. The samples were placed on the bottom of small plastic Petri dishes (5 cm diameter; Conthey, Reckenholz, Wädenswil) or plastic cylinders (10 cm diameter; Changins, Frick) and enclosed in a metal grid (1.5 cm diameter mesh size) to exclude larger predators such as mice. One sample was clamped between two branches at a height of 1.5 to 1.8 m and the two others were installed on the ground. One of the latter two served as a control and was protected from predation by a ventilated plastic jar (0.8 L). Care was taken to place the samples in locations protected from rain and direct sunlight. The samples were exposed in the hedges for four days, but the period was prolonged or shortened on a few occasions to two or five days in the case of cold weather, and thus low predator activity, or to avoid the emergence of adult flies when high temperatures prevailed. After field exposure, the samples were collected and the pupae were counted and classified into: Intact pupae, damaged pupae and missing pupae. The samples were then put into plastic jars and transferred into climate chambers to check for the capacity of collected pupae to complete their development and allow adults to emerge.

### 2.3. Collection of Arthropod Predators

Predators were captured at each location in both hedges at several meters distance from the exposed pupae at intervals of about one month between June and October 2019 (Table 1). Predators were collected by three different trap types: pitfall traps, i.e., vertically buried soil traps filled with vegetal material (a 10 cm diameter PVC tube completed with a plastic bottle (200 mL) and a funnel connecting the top of the soil with the top of the bottle, two traps per hedge), bamboo pipes (0.5–1.0 cm diameter and 10–15 cm long, five pipes per hedge), and corrugated cardboard strips (band of 2 × 30 cm wrapped around a branch, five strips per hedge). Traps were inspected 12 and 24 h after deployment and then removed until the following scheduled collection. Additionally, predators were collected by beating the shrubs and gathering the fallen arthropods from a net placed below. Approximately 15–30 min of beating were invested per hedge and sampling date. Each predator was directly trapped into an Eppendorf tube (1.5 mL) and put on ice until reaching the laboratory where the samples were stored at −20 °C. Predators were morphologically determined to family or order level while being kept on crushed ice. Spiders were imaged with a high-resolution 4K numeric microscope camera, series VHX-7000 (Keyence, Osaka, Japan) and determined to family level using the photographs.

### 2.4. Molecular Gut Content Analysis

Field sampled predators were subjected to analyses using a PCR-based approach based on the Dro-suz-S390 and Dro-suz-A380 primers (Eurofins Genomics, Ebersberg, Germany), and a modified protocol based on Wolf et al. [22]. Although the whole body of the predator was used in the analyses, we refer to common terminology and use the term molecular gut content analyses for this approach. The used primers specifically target a 171 bp sequence of the COI gene from *D. suzukii*. To avoid any contamination of the predators’ body surface with *D. suzukii* DNA, samples were washed with bleach and water prior to analysis [27,28]. To each 1.5 mL tube containing a single predator, 1 mL bleach 1–1.5% solution (1 part 10–15 % sodium hypochlorite (VWR Chemicals, Radnor, PA, USA) 9 parts Milli-Q water, and Tween 20 (0.1%) was added and the tubes were then shaken for 30 s. They were then rinsed twice with 1 mL Milli-Q water and shaken for another 15 s.

#### 2.4.1. DNA-Extraction

The whole predator body was added to 180 μL TES buffer (0.1 M TRIS, 10 mM EDTA, 2% SDS, pH 8) and 20 μL Proteinase K (20 mg/mL) and crushed manually with sterilized pipette tips. The samples were then centrifuged for 5 min at 17,000× *g* and incubated overnight on a rocking platform (500 rpm; Hettlich Benelux, Geldermalsen, The Netherlands) at 56 °C. DNA was extracted with the DNeasy blood & tissue Kit (Qiagen, Hilden, Germany) following the manufacturer’s instructions, except for the lysis step described above and the elution with 1× TE buffer. To determine the samples’ DNA concentrations, the Nanodrop 1000 Spectrophotometer (Thermo Fischer Scientific, Waltham, MA, USA) was used. Each sample was adjusted to the same concentration of DNA (5 ng/μL) using the PIPETMAX^®^ device (Gilson, Lewis Center, OH, USA).

#### 2.4.2. PCR

The COI target sequence was amplified from all samples using the Tap PCR Core Kit (Qiagen, Hilden, Germany): 1 μL reaction buffer (10×), 0.2 μL dNTP mix (10 mM each), 0.1 μL MgCl_2_ (25 mM), 0.2 μL primer Dro-suz-S390 (10 μM), 0.2 μL primer Dro-suz-A380 (10 μM), 0.025 μL Taq polymerase (250 Units), 0.5μL BSA (10 mg/mL), and 5.775 μL of sterilized, filtered, and UV exposed H_2_0 were prepared and poured in each well, followed by the addition of 2 μL of the corresponding DNA template. The 10 μL PCR samples were processed by the PCR C1000 Touch Thermal Cycler (BioRad, Hercules, California, USA) with the following conditions: Initial denaturation for 15 min at 95 °C, followed by 35–40 cycles of 30 s at 94 °C, 90 s at 62 °C, 60 s at 72 °C and a final extension for 10 min at 72 °C.

#### 2.4.3. Gel Electrophoresis

To determine presence of *D. suzukii* in a sample, 3 μL of the PCR product were analyzed on a 2% agarose gel. Visualization of the PCR product was performed with a UV analyser Quantum (Vilber, Collégien, France). A sample was defined as containing *D. suzukii* DNA if the PCR product of 171 bp was visible. To obtain a positive control, 5 earwigs were collected in the field and then kept individually in petri dishes (5 cm) for 24 h with a humid cotton pad only. The next day, they were fed two *D. suzukii* pupae and were observed during 3 h. Finally, their gut content was analyzed as described above. The DNA isolated from an earwig that had eaten both *D. suzukii* pupae and thus produced a strong band on the gel was kept and used as a positive control. Water instead of DNA was added for the negative control. To exclude false-positives, all positive samples were re-analyzed. Only samples that tested positive twice were considered to have fed *D. suzukii*.

### 2.5. Statistical Analyses

The proportion of predated pupae and the number of predators per group were analyzed with generalized linear mixed models using the R-package lme4 [29] in R version 4.0.1. [30] The model for the analysis of the proportion of predated pupae used binomial-distributed errors, the model for the number of predators used Poisson-distributed errors. The models used the fixed effects hedge type (dry and humid) and exposure period (1–4). The model analyzing predation further included the fixed effects exposure place (control, ground and branch) and exposure time (2–5 days). The model analyzing the number of predators additionally included the fixed effect predator group. Non-significant interactions and the non-significant variable exposure time and its quadratic term were removed from the models in a stepwise process. The random effect location explained a significant proportion of the variance only in the model for the analysis of the proportion of predated pupae. A random effect observational level was included in both models because of overdispersion. For better convergence, the models used the optimizer “bobyqa” instead of “Nelder-Mead” for the second phase. Tukey post-hoc multiple comparisons were performed using the R-package lsmeans [31]. Model assumptions were validated.

The composition of predator groups and Araneae families was analyzed with transformation-based redundancy analyses with the R package vegan [32]. Data was Hellinger transformed. The models used the describing variables hedge type (dry and humid) and sampling period (1–4) and the random effect location. Because of unbalanced data from different dates and sites, permutation tests used type III sum of squares. The number of permutations was 999.

## 3. Results

### 3.1. Exposure of D. suzukii Pupae in the Field

Overall 2113 pupae of *D. suzukii* were exposed to predation in the hedges. Adults emerged from 33.1 ± 4.0% of them after taking them back to the laboratory, and 43.7 ± 4.1% were considered to be predated (pupae were missing or damaged) while the fate of 23.2 ± 3.1% remained unaccounted for (pupae were returned visibly undamaged but no adult flies emerged; Table 2). From the 1090 control pupae, 67.4 ± 4.9% yielded adult flies, while 1.9 ± 1.1% were lost and 29.6 ± 4.7% remained unaccounted for. Mean predation over the whole assessment period varied largely among hedges and ranged from 6.7 ± 3.8% on the ground at the humid site in Wädenswil to 70.0 ± 21.3% on branches at the dry site in Changins.

**Figure 1:**
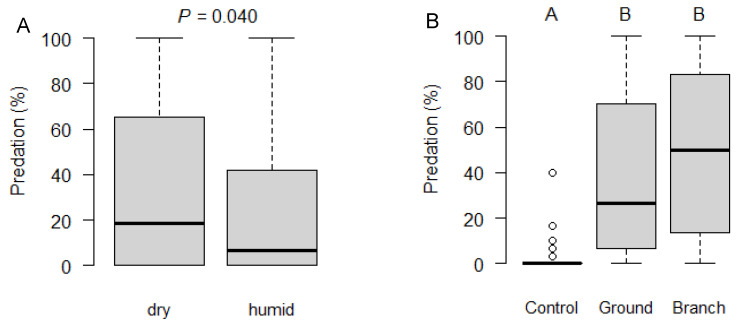
Percent predation on pupae of *Drosophila suzukii* exposed during 2–5 days in 10 hedges across Switzerland. Predation according to (**A**) type of hedge and (**B**) according to exposure site. Different letters indicate significant differences (Tukey multiple comparisons: *P* < 0.05). The solid line indicates the median, the box goes from the first to the third quartile, whiskers indicate 1.5 × the interquartile distance and circles outliers.

The proportion of predated pupae was significantly higher at dry than at humid sites (*χ^2^*_1,103_ = 4.206, *P* = 0.040; Figure 1A). Furthermore, predation significantly differed between exposure sites (*χ^2^*_2,103_ = 67.295, *P* < 0.001): predation on pupae exposed on the ground (*z* = 6.965, P < 0.001) or on branches (*z* = 7.871, *P* < 0.001) was significantly higher as compared to the control pupae kept in enclosed plastic jars (Figure 1B). However, predation on pupae exposed on branches or the ground did not differ significantly (*z* = 1.215, *P* = 0.444). The proportion of predated pupae did not significantly vary between sampling periods (χ^2^_1,103_ = 1.915, *P* = 0.590).

### 3.2. Collection of Arthropod Predators

**Figure 2:**
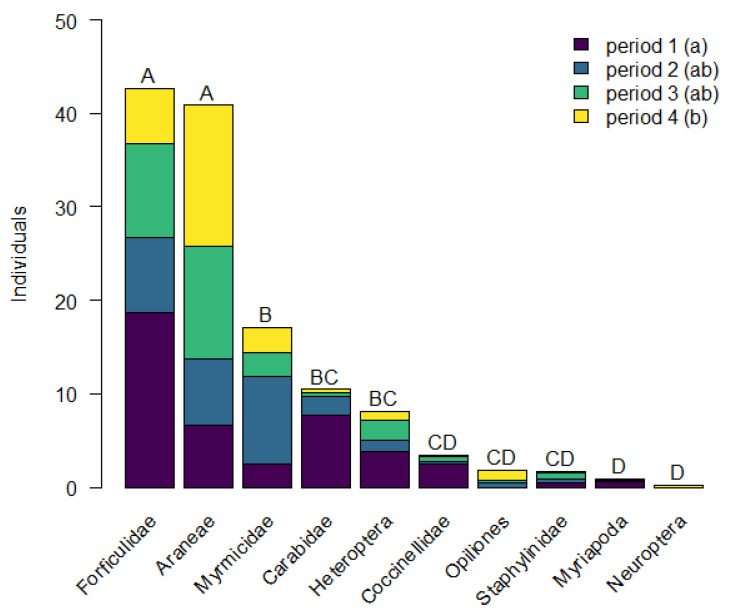
Mean number of individuals per sampling period (period 1: 17 June–4 July; period 2: 8 July–25 July; period 3: 12 August–4 September; period 4: 17 September–16 October) and predator group. Different letters indicate significant differences (Tukey multiple comparisons: *P* < 0.05) between levels of the different predator groups (A–D, above the bars) and sampling periods (a–b, in brackets).

In total, 1101 predator individuals were collected. The number of predators differed significantly between groups (*χ^2^*_9,345_ = 178.298, *P* < 0.001; Figure 2). The highest numbers of arthropods collected were insects of the families Forficulidae (357), Myrmicidae (160) and Carabidae (74), as well as 383 spiders of the main families Tetragnathidae (61), Thomisidae (58), Philodromidae (34), and Anyphaenidae (34). The number of collected predators differed significantly between sampling periods (*χ^2^*_3,345_ = 10.504, *P* = 0.015), as more predators were collected in the first sampling period than in the last (*z* = 3.206, *P* = 0.007). The number of predators did not differ significantly between dry and humid hedges (*χ^2^*_1,345_ = 0.086, *P* = 0.769). Hedge type (*F*_1_ = 1.565, *P* = 0.158; Figure S1A) and sampling period (*F*_3_ = 1.692, *P* = 0.053; Figure S1B) only explained 15.0% of the variance in the composition of predator groups. Alike, hedge type (*F*_1_ = 1.746, *P* = 0.065; Figure S2A) and sampling period (*F*_1_ = 1.480, *P* = 0.071; Figure S2B) only explained 23.0% of the variance in the composition of Araneae families.

### 3.3. Molecular Gut Content Analyses

Out of 1101 predators, 20 (=1.8%) tested positive to *D. suzukii* DNA; among them 12 (=3.4%) Forficulidae, 7 (=1.8%) Araneae and 1 (=1.6%) Heteroptera. Among the positive tested Forficulidae, one was collected in the dry hedge in Frick (25.06), four in the dry and two in the humid hedge in Conthey (all 16.07, except one in the dry hedge on 27.08), each two in the dry and humid hedge in Changins (all 26.8 except one in the humid hedge on 22.07) and one in the humid hedge in Wädenswil (04.09). Two positive tested Araneae were collected in the dry hedge in Frick (a Thomisidae on 25.06 and a Pisauridae on 18.9), one in the humid (a Clubionidae on 03.10) and all others in the dry hedge in Wädenswil (each one Thomisidae on 04.09 and 03.10 and two Dictynidae on 3.10). The Heteroptera was collected on 04.09 in the humid hedge in Wädenswil.

The proportion of collected predators that were tested positive for the presence of *D. suzukii* DNA over the four sampling periods was 0.78% in period 1 (17.06–04.07; n = 258; positive 2), 2.08% in period 2 (08.07–25.07; n = 288; positive 6), 2.44% in period 3 (12.08–04.09; n = 287; positive 7), and 1.87% in period 4 (17.09–16.10; n = 268; positive 5). When only Heteroptera, Araneae, and Forficulidae were considered, the percentage of *D. suzukii* positive individuals ranged from 1.14 to 3.70. Although the percentage of positive individuals was lowest during the first sampling period, no considerable differences could be identified over the four sampling periods and the proportion of positive predators did not change over the season.

## 4. Discussion and Conclusions

Our field survey at five different locations in two types of hedges showed that, on average, 44% of the exposed *D. suzukii* pupae were predated with few incidences of up to 95% of the provided pupae unrecovered. Predation took place on the ground as well as in the branches and across all sampling periods. However, although several families of predatory arthropods were present in the hedges and more than thousand individuals were collected and analyzed, less than 2% of the captured predators tested positive for the *D. suzukii* COI marker and only earwigs, spiders, and one predatory bug were identified to have recently fed on this invasive pest species.

Although web spiders may mainly capture adult flies and predatory bugs mainly feed on eggs, immobile pupae are probably the life stage of *D. suzukii* most vulnerable to predation [22,24,33]. Developing eggs and larvae are well hidden within the fruits, whereas pupae often protrude through the skin of the fruit or are found on the ground. Even though pupae might have been more exposed and aggregated in our study than under natural conditions, the observed predation rate of 44% of exposed pupae is considerable and likely affects the fly’s population dynamic. The calculated predation rate largely exceeds OECD requirements of a 10% reduction in the pest population for the registration of an invertebrate biocontrol agent [34]. Moreover, the calculated rates of predation are in accordance with previous studies. Woltz and Lee [35] observed a decrease of 61–91% in the number of recovered pupae exposed on the soil in strawberry, blueberry, and blackberry fields. Similarly, Ballman et al. [36] found predation rates on exposed pupae from 34 to up to 100% in wild blueberry fields. Considering all three studies, it seems that generalist predators might have a higher impact on population dynamics of *D. suzukii* in Europe and North America than the more specialized parasitoids, whose natural parasitism rates rarely exceed 10 % [33,37,38]. Since parasitoids in the invaded areas mainly attack pupae, an additive effect of the two guilds of natural enemies is plausible [39,40].

We observed a high spatial variability in predation between hedges at the five locations, with calculated mean predation rates ranging from 6.7 ± 3.8% on the ground of a humid hedge to 70.0 ± 21.3% on the branches of a dry hedge. Significantly more *D. suzukii* pupae were predated in dry than humid hedges, but there was no significant difference in predation between pupae that were exposed on the ground and on branches. Likewise, predation did not significantly vary among the four sampling periods. These findings indicate that dry and sunny hedges harbor more voracious or higher densities of pupal predators than wet and shady hedges. Overall, dry hedges also hosted a higher plant diversity (see Table 1), which might thereby provide more food and alternative prey to generalist predators [21]. Accordingly, a general relationship between plant diversity and predation also became visible. In Conthey and Frick, where humid hedges showed a high plant diversity, higher predation in humid hedges was also observed, whereas in Changins and Wädenswil, the lower plant diversity in humid hedges was accompanied by less predation. In addition, pupal predators on the ground and on branches were of comparable voracity or even consisted of similar complexes of species. The latter might be the case, since in particular ants and earwigs are assumed to be important predators of *D. suzukii* pupae [22,24,25,35,41]. Typically, they live and forage on the ground as well as in the canopy and both are probably able to remove pupae entirely [35], which could explain the missing 34.4 ± 3.6% of the exposed pupae. Furthermore, ants and earwigs are present and active over the whole vegetative season, which might further explain the lack of significant differences between exposure periods. Yet, the presence of the *D. suzukii* COI marker could only be confirmed in the gut of earwigs but not in any one of the 160 collected ants. Since ants usually carry captured prey to their colonies where it is fed to larvae and shared among its members, it might, however, be difficult to actually detect any *D. suzukii* DNA in ants.

Earwigs, ants, spiders, and ground beetles were the most collected predator taxa. The composition of the predator communities and their abundance did not significantly differ between hedge types. Similarly, hedge type did not significantly influence the taxonomic composition of spiders at the family level. It can be assumed that the rather coarse identification up to the genus or family level, as was limited by the sample preservation for molecular analyses, does not permit to find more subtle habitat-specific differences at the species level. Although predator abundance was significantly higher in June to early July, as compared to late September to October; species composition of predator groups and Araneae families did not vary between sampling periods. The former could be due to warmer temperatures in summer than in fall, which is similar to the higher predation rates found in dry and warm hedgerows compared to humid hedges.

In the 1,101 captured predators, the presence of the *D. suzukii* COI marker could be confirmed in fewer than 2% of the tested individuals and only in the three orders Forficulidae, Araneae, and Heteroptera. The proportion of individuals that had fed on *D. suzukii* was highest in earwigs, whereas fewer proportions tested positive among spiders and bugs. The same predator orders, and furthermore rove beetles (Staphylinidae), have also been identified to feed on *D. suzukii* in the study by Wolf et al. [22]. The proportion of predators that tested positive for the *D. suzukii* COI marker was, however, considerably higher in the study of Wolf et al. [22] with 43.4% for earwigs and around 15 % for spiders and bugs. This might in part be explained by higher *D. suzukii* density in the study of Wolf et al. [22], in which arthropod predators have been captured in the immediate vicinity of heavily infested cherries, blackberries, and raspberries, whereas we also collected many specimens on non-fruiting plant species. In order to prevent effects of volatiles on the predators and to avoid reducing prey availability, we deliberately refrained from setting up *D. suzukii* traps in the monitored hedges. We therefore have no observational data on the effective abundance of *D. suzukii* in the studied hedges. Furthermore, semi-natural habitats are known to harbor alternative prey to generalist predators [21]. Thus, *D. suzukii* density might have been lower in our study, while alternative prey was more common, which probably reflects the natural pattern of predation in agricultural hedges. 

The proportion of predators that was tested positive for the *D. suzukii* COI marker was surprisingly low in comparison to the observed predation rate. It is likely that we did not cover the full range of predators in our molecular analyses, either because they carried away the prey such as ants or because they were not analyzed, such as predatory flies or mollusks. Furthermore, the relatively small proportion of positive samples can be explained by the detection limits of the analysis. Molecular gut content analyses allow the detection of prey DNA before it is digested to below the detection limit, which usually takes between several hours to a few days [42]. Under laboratory conditions, the DNA of *D. suzukii* was detectable in the gut of the common earwig for up to 48 h [43]. Moreover, we also decided to use a conservative approach to effectively validate the presence of *D. suzukii* DNA in the gut of predators, since individuals had to be tested positive twice in independent PCR runs. Hence, we might have dismissed samples where a very small amount of *D. suzukii* DNA failed to be confirmed in the repetition. It can therefore be assumed that our molecular assay underestimated the proportion of predators, which had fed on *D. suzukii* pupae.

Overall, a rather high proportion of exposed pupae was preyed by generalist predators in the studied hedgerows. Due to their broad prey range, the identified predators might however be less suited for targeted biological control attempts compared to more specialist parasitoid species from *D. suzukii*’s native range or even from invaded regions [44]. Furthermore, high densities of some predators like the omnivorous earwigs can also inflict damage to thin-skinned fruits such as cherries or grapes [45,46,47]. Nonetheless, these generalist predators might provide a non-negligible background regulation of *D. suzukii* and their presence in hedgerows in the agricultural landscape may contribute to the regulation of pest populations [33,48]. Differences in composition of the predator community and the predation of pupae between sites were high, indicating that landscape context and hedge quality affect the extent of biological control provided by natural enemies, and suggesting a still underexploited potential to improve natural pest regulation. However, whether an individual hedge functions as a sink or a source for *D. suzukii* may also depend on the community of host plant species. For example, plants from the genus *Cornus*, *Prunus*, *Rubus*, or *Sambucus* are attractive and favorable to *D. suzukii* [4] and probably enhance its spillover from hedges to nearby crops (e.g.,: [10,11,49,50]). Hedges with less suited plant species or even harboring dead-end hosts (i.e., fruits that are used for oviposition but do not support larval development) might however function as sinks [5,51,52]. Moreover, hedgerows might provide other ecosystem services such as wind breaks, the conservation of biodiversity, the control of other pests, the supply of edible fruits, and attractiveness for recreation, thereby enhancing the overall quality of agricultural landscapes.

## Figures and Tables

**Table 1:** Locations, exposure periods of *Drosophila suzukii* pupae, sampling dates, and plant species of hedges in five different locations in Switzerland. Exposure and sampling periods are followed in brackets by the scaling level for statistical analysis. VD: Vaud; VS: Valais; AG: Aargau; ZH: Zurich.

Location (Canton)	Exposure Period	Sampling Predators	Hedge Type	Lat. [N]/ Lon. [E]	Host Plant Species
Changins (VD)	1.7–4.7 (1) 22.7–25.7 (2) 27.8–30.8 (3) 3.10–6.10 (4)	1.7–4.7 (1) 22.7 (2) 26.8–27.8 (3) 30.9 (4) 3.10–4.10 (4)	dry	46°24′09.1″/ 6°13′42.4″	*Cornus sanguinea*, *Euonymus europaeus*, *Hedera helix*, *Lonicera xylosteum*, *Prunus avium*, *P. spinosa*, *Rosa* spp., *Rubus* spp., *Sambucus nigra*, *Viburnum lantana*, *V. opulus*
			humid	46°23′47.3″/ 6°13′42.0″	*Bryonia dioica*, *Cornus sanguinea*, *Rubus* spp., *Sambucus nigra*
Conthey (VS)	16.7–18.7 (2) 12.8–15.8 (3) 4.10–7.10 (4)	16.7 (2) 12.8–13.8 (3) 27.8 (3) 2.10–4.10 (4)	dry	46°12′37.6″/ 7°18′02.6″	*Cornus* spp., *Sambucus nigra*, *Viburnum lantana*, *V. opulus*
			humid	46°14′07.5″/ 7°18′35.7″	*Hipophae rhamnoides*, *Prunus avium*, *P. cerasifera*, *P. mahaleb*, *Rosa* spp., *Sambucus nigra*
Frick (AG)	24.6–28.6 (1) * 21.7–26.7 (2) 13.8–16.8 (3) 17.9–20.9 (4) 15.10–18.10 (4)	25.6 (1) 23.7–25.7 (2) 12.8–14.8 (3) 17.9, 18.9 (4) 16.10 (4)	dry	47°30′58.3″/ 8°1′26.6″	*Amelanchier ovalis*, *Cornus* spp., *Crataegus* spp., *Euonymus europaeus*, *Ligustrum* spp., *Lonicera xylosteum*, *P. spinosa*, *Rhamnus frangula*, *Rosa* spp., *Rubus* spp., *Sambucus nigra*, *Viburnum opulus*
			humid	47°30′58.28″/ 8°1′26.62″	*Cornus* spp., *Crataegus* spp., *Euonymus europaeus*, *Ligustrum* spp., *Prunus spinosa*, *Rhamnus frangula*, *Rosa* spp., *Rubus* spp., *Sambucus nigra*, *Viburnum opulus*
Reckenholz (ZH)	25.6–28.6 (1) 8.7–12.7 (2) 22.8–26.8 (3) 4.10–8.10 (4)	17.6. (1) 8.7–9.7 (2) 22.8 (3) 4.10 (4)	dry	47°25′34.9″/ 8°31′02.4″	*Cornus sanguinea*, *Crataegus* spp., *Ligustrum vulgare*, *Lonicera xylosteum*, *Rosa canina*, *Rhamnus cathartica*
			humid	47°25′32.9″/ 8°31′18.8″	*Crataegus* spp., *Prunus spinosa*
Wädenswil (ZH)	5.7–8.7 (2) 30.8–2.9 (3) * 1.10–4.10 (4)	9.7 (2) 4.9 (3) 3.10–4.10 (4)	dry	47°21′8.11″/ 8°68′24.2″	*Amelanchier ovalis*, *Cornus* spp., *Crataegus* spp., *Euonymus europaeus*, *Hedera helix*, *Ligustrum vulgare*, *Prunus padus*, *P. spinosa*, *Rhamnus cathartica*, *Rosa* spp., *Rubus* spp., *Sambucus nigra*, *S. racemosa*, *Taxus baccata*, *Viburnum opulus*
			humid	47°22′37.5″/ 8°67′59.3″	*Cornus* spp., *Hedera helix*, *Ligustrum vulgare*, *Rubus* spp., *Sambucus nigra*

* Only 15 instead of 30 pupae were exposed due to rearing limitations.

**Table 2:** Detailed results for all 10 hedges in different locations in Switzerland, where *Drosophila suzukii* pupae were exposed for predation. D: dry; H: humid, SE: standard error.

	Changins		Conthey		Frick		Reckenholz		Wädenswil	
	D	H	D	H	D	H	D	H	D	H
Number exposed	210	243	180	190	270	270	240	240	150	120
Number predated	114	27	77	87	109	124	152	144	78	17
Mean predation [%]	54.3	10.7	42.8	47.5	40.3	46.0	63.3	60.0	52.2	12.7
SE predation	16.6	6.4	15.4	12.8	10.7	10.5	6.3	9.9	21.4	8.1
Mean unaccounted [%]	18.1	25.6	12.2	20.8	45.0	46.3	15.4	8.8	6.7	7.3
SE unaccounted	10.7	6.4	5.0	6.0	11.5	11.4	3.0	1.8	4.8	3.1
Samples	7	8	6	6	10	10	8	8	6	5

## Data Availability

The raw data for this study are available at: https://doi.org/10.6084/m9.figshare.14096693.v1 (accessed on 29 March 2021).

## Supplementary Materials

The following are available online at https://www.mdpi.com/article/10.3390/insects12040305/s1, Figure S1: Transformation-based redundancy analyses of the composition of predator groups in dry and humid hedges and over four sampling periods, Figure S2. Transformation-based redundancy analyses of the composition of Araneae families in dry and humid hedges and over four sampling periods.
