# Supplementary material for: Predation on Drosophila suzukii within Hedges in the Agricultural Landscape
Source: Insects. 2021 Mar 30;12(4):305. doi: 10.3390/insects12040305 (PMC8067151; doi:10.3390/insects12040305)
Supplement: Supplementary file 1 [file insects-12-00305-s001.pdf]

## Supplemental Figures

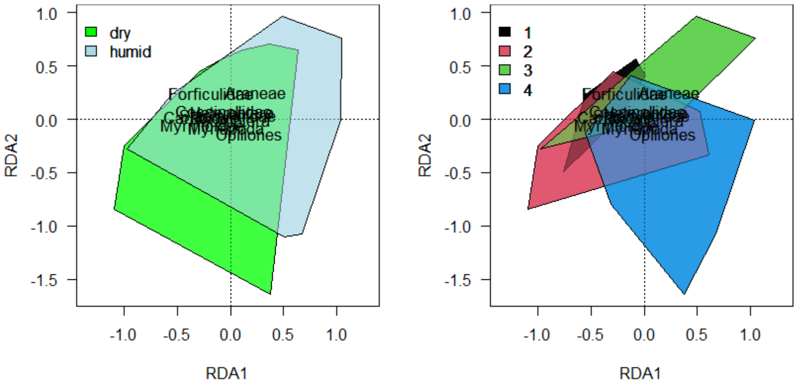


**Figure S1.** Transformation-based redundancy analyses (999 permutations) of the composition of predator groups in dry (green) and humid (blue) hedges ($F_1$ = 1.565, $P$ = 0.158; left plot) and over four sampling periods ($F_3$ = 1.692, $P$ = 0.053; right plot).

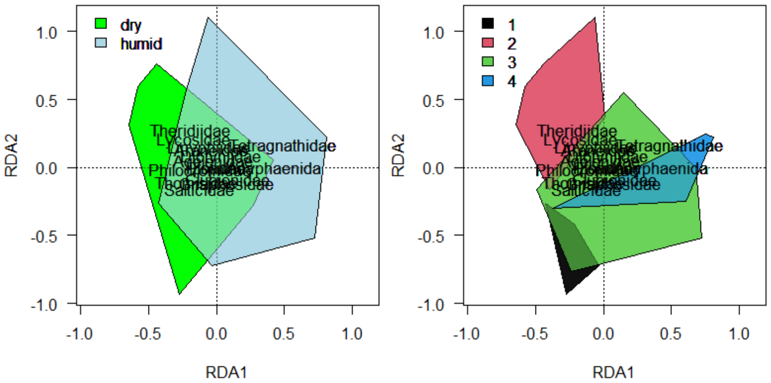


**Figure S2.** Transformation-based redundancy analyses (999 permutations) of the composition of Araneae families in dry (green) and humid (blue) hedges ($F_1$ = 1.746, $P$ = 0.065; left plot) and over four sampling periods ($F_1$ = 1.480, $P$ = 0.071; right plot).
